# An unsupervised classification scheme for improving predictions of prokaryotic TIS

**DOI:** 10.1186/1471-2105-7-121

**Published:** 2006-03-09

**Authors:** Maike Tech, Peter Meinicke

**Affiliations:** 1 Abteilung Bioinformatik, Institut für Mikrobiologie und Genetik, Georg-August-Universität Göttingen, Goldschmidtstr. 1, 37077 Göttingen, Germany

## Abstract

**Background:**

Although it is not difficult for state-of-the-art gene finders to identify coding regions in prokaryotic genomes, exact prediction of the corresponding translation initiation sites (TIS) is still a challenging problem. Recently a number of post-processing tools have been proposed for improving the annotation of prokaryotic TIS. However, inherent difficulties of these approaches arise from the considerable variation of TIS characteristics across different species. Therefore prior assumptions about the properties of prokaryotic gene starts may cause suboptimal predictions for newly sequenced genomes with TIS signals differing from those of well-investigated genomes.

**Results:**

We introduce a clustering algorithm for completely unsupervised scoring of potential TIS, based on positionally smoothed probability matrices. The algorithm requires an initial gene prediction and the genomic sequence of the organism to perform the reannotation. As compared with other methods for improving predictions of gene starts in bacterial genomes, our approach is not based on any specific assumptions about prokaryotic TIS. Despite the generality of the underlying algorithm, the prediction rate of our method is competitive on experimentally verified test data from *E. coli *and *B. subtilis*. Regarding genomes with high G+C content, in contrast to some previously proposed methods, our algorithm also provides good performance on *P. aeruginosa*, *B. pseudomallei *and *R. solanacearum*.

**Conclusion:**

On reliable test data we showed that our method provides good results in post-processing the predictions of the widely-used program GLIMMER. The underlying clustering algorithm is robust with respect to variations in the initial TIS annotation and does not require specific assumptions about prokaryotic gene starts. These features are particularly useful on genomes with high G+C content. The algorithm has been implemented in the tool »TICO«(TIs COrrector) which is publicly available from our web site.

## Background

Recent publications have shown that gene prediction in prokaryotes is still a challenging problem in bioinformatics. While existing gene finders [1-3] are likely to identify coding regions associated with open reading frames (ORF) of statistically significant length, the prediction of the true translation initiation sites (TIS) is insufficient in many cases [4-6]. In particular, for genomes with a high G+C content the prediction of TIS has been shown to be of low quality [6].

In order to cope with this insufficiency of conventional gene finders, several methods have been proposed for improving the predictions of prokaryotic TIS. Common approaches require prior knowledge about the characteristics of the ribosome binding site (RBS) as achieved by analysis of the 16SrRNA or by some supervised method for inferring model parameters from sequences of known gene starts [7-9]. In contrast, more recent methods focus on unsupervised schemes for optimizing most of the TIS related parameters [5,4,10,2] without any examples of known TIS. Therefore, in principle no prior knowledge about the RBS or TIS is needed. But it is important to note that these methods usually include TIS related parameters that cannot be optimized by some unsupervised algorithm. For example, the length of the RBS or the spacer between RBS and translation start have been used as parameters (see section »Comparison with other tools for improvement of TIS predictions« for a detailed discussion). In order to adjust these parameters in an optimal way, again prior knowledge would be necessary.

We recently introduced the tool »TICO« (TIs COrrector) for improving the results of conventional gene finders. TICO is based on a very general model which does not involve any parameters for representing specific characteristics of translation starts. In a previous publication [11] we described the web interface for TICO and presented results on the genome of *Escherichia coli *K-12. In the present paper we give a detailed description of the underlying algorithm and we provide a thorough evaluation of the method on a significantly enlarged set of genomes. In particular, we present a detailed comparison with three other tools for improving predictions of prokaryotic TIS. In addition, we present an extension of the algorithm for automatic adaptation of a smoothing parameter which had to be specified by the user in the first version of »TICO«. The results indicate that, despite its generality, our algorithm yields overall good performance, which also involves predictions on the high-G+C genomes we tested.

### Unsupervised classification of TIS sequences

Our TIS prediction method is based on a clustering algorithm, which assigns candidate TIS sequences to one of two classes for representation of *strong *and *weak *candidates, respectively. Each of the two classes is represented by an inhomogeneous second order probability model. The position-dependent probabilities are estimated from the trinucleotide occurrences in the proximity of potential start sites. In order to cope with vanishing probabilities, we apply positional smoothing [12] to the probabilities, as detailed in the next section.

In an initial step, the putative start positions, as predicted by a conventional gene finder, are labeled as *strong *TIS candidates. Additional candidates associated with potential start codons are obtained from a search range around these initial positions. The additional sites are labeled as *weak *TIS candidates. As a constraint, all candidate TIS for one ORF should share the same reading frame, and no stop codon is allowed to occur between a potential start and the annotated stop.

Starting with the initial classification we iterate the following two successive steps:

#### 1. Estimation

Trinucleotide probability models for the *strong *and *weak *categories are estimated from all *strong *and *weak *TIS sequences, respectively. We apply positional smoothing to the trinucleotide probabilities using a discretized Gaussian density function (see following section). The TIS sequences are extracted as the flanking regions of the potential start codons with a fixed number of upstream and downstream positions. Finally, a second order positional weight matrix (PWM) is built from the probabilities by subtracting the logarithms of the position specific *weak *model probabilities from the *strong *ones.

#### 2. Classification

The PWM is used to score all TIS candidates. The candidate with highest positive score among all candidates of the same gene-specific search range is classified as *strong *TIS; all other candidates from that range are classified as *weak *TIS. This implies the following special cases: if there is no range-specific candidate with a positive score, all candidates of the corresponding range are classified as *weak *TIS; if there are several range-specific candidates with a positive score, only that candidate with maximum score is labeled *strong*.

The two steps of estimation and classification are iterated until the classification does not change anymore or a maximum number of 20 iterations has been reached. The total set of candidates associated with the ORF-specific search ranges does not change during the iterations. The resulting candidates with maximum score from the corresponding ranges are considered as the final TIS predictions of the algorithm.

It is not difficult to see that the above clustering algorithm must converge in terms of a monotonically increasing total sum of scores. To see this, we can formulate an objective function for the clustering using binary assignment variables *h*_*ij *_∈ {0, 1}, which classify the *j*-th candidate TIS of the *i*-th putative gene according to *strong *(*h*_*ij *_= 1) or *weak *(*h*_*ij *_= 0) categories, respectively. In addition, we can represent the models by matrices **P**_*s *_and **P**_*w *_with entries pkls
 MathType@MTEF@5@5@+=feaafiart1ev1aaatCvAUfKttLearuWrP9MDH5MBPbIqV92AaeXatLxBI9gBaebbnrfifHhDYfgasaacH8akY=wiFfYdH8Gipec8Eeeu0xXdbba9frFj0=OqFfea0dXdd9vqai=hGuQ8kuc9pgc9s8qqaq=dirpe0xb9q8qiLsFr0=vr0=vr0dc8meaabaqaciaacaGaaeqabaqabeGadaaakeaacqWGWbaCdaqhaaWcbaGaem4AaSMaemiBaWgabaGaem4Camhaaaaa@3271@ and pklw
 MathType@MTEF@5@5@+=feaafiart1ev1aaatCvAUfKttLearuWrP9MDH5MBPbIqV92AaeXatLxBI9gBaebbnrfifHhDYfgasaacH8akY=wiFfYdH8Gipec8Eeeu0xXdbba9frFj0=OqFfea0dXdd9vqai=hGuQ8kuc9pgc9s8qqaq=dirpe0xb9q8qiLsFr0=vr0=vr0dc8meaabaqaciaacaGaaeqabaqabeGadaaakeaacqWGWbaCdaqhaaWcbaGaem4AaSMaemiBaWgabaGaem4DaChaaaaa@3279@, indicating the probability of trimer *k *on position *l *for the *strong *and *weak *models, respectively. For gene *i *the sequence of the *j*-th candidate TIS can be represented by a matrix **X**_*ij *_with binary entries xklij
 MathType@MTEF@5@5@+=feaafiart1ev1aaatCvAUfKttLearuWrP9MDH5MBPbIqV92AaeXatLxBI9gBaebbnrfifHhDYfgasaacH8akY=wiFfYdH8Gipec8Eeeu0xXdbba9frFj0=OqFfea0dXdd9vqai=hGuQ8kuc9pgc9s8qqaq=dirpe0xb9q8qiLsFr0=vr0=vr0dc8meaabaqaciaacaGaaeqabaqabeGadaaakeaacqWG4baEdaqhaaWcbaGaem4AaSMaemiBaWgabaGaemyAaKMaemOAaOgaaaaa@33CA@ ∈ {0, 1} indicating the occurrence of trimer *k *at position *l*. With these definitions the objective of the clustering algorithm is:

maximize

F(Ps,Pw,{hij})=∑i,jhij∑k,lxklijlog⁡pkls+∑i,j(1−hij)∑k,lxklijlog⁡pklw     (1)
 MathType@MTEF@5@5@+=feaafiart1ev1aaatCvAUfKttLearuWrP9MDH5MBPbIqV92AaeXatLxBI9gBaebbnrfifHhDYfgasaacH8akY=wiFfYdH8Gipec8Eeeu0xXdbba9frFj0=OqFfea0dXdd9vqai=hGuQ8kuc9pgc9s8qqaq=dirpe0xb9q8qiLsFr0=vr0=vr0dc8meaabaqaciaacaGaaeqabaqabeGadaaakeaacqWGgbGrcqGGOaakieqacqWFqbaudaWgaaWcbaGaem4CamhabeaakiabcYcaSiab=bfaqnaaBaaaleaacqWG3bWDaeqaaOGaeiilaWIaei4EaSNaemiAaG2aaSbaaSqaaiabdMgaPjabdQgaQbqabaGccqGG9bqFcqGGPaqkcqGH9aqpdaaeqbqaaiabdIgaOnaaBaaaleaacqWGPbqAcqWGQbGAaeqaaaqaaiabdMgaPjabcYcaSiabdQgaQbqab0GaeyyeIuoakmaaqafabaGaemiEaG3aa0baaSqaaiabdUgaRjabdYgaSbqaaiabdMgaPjabdQgaQbaaaeaacqWGRbWAcqGGSaalcqWGSbaBaeqaniabggHiLdGccyGGSbaBcqGGVbWBcqGGNbWzcqWGWbaCdaqhaaWcbaGaem4AaSMaemiBaWgabaGaem4CamhaaOGaey4kaSYaaabuaeaacqGGOaakcqaIXaqmcqGHsislcqWGObaAdaWgaaWcbaGaemyAaKMaemOAaOgabeaakiabcMcaPaWcbaGaemyAaKMaeiilaWIaemOAaOgabeqdcqGHris5aOWaaabuaeaacqWG4baEdaqhaaWcbaGaem4AaSMaemiBaWgabaGaemyAaKMaemOAaOgaaOGagiiBaWMaei4Ba8Maei4zaCMaemiCaa3aa0baaSqaaiabdUgaRjabdYgaSbqaaiabdEha3baaaeaacqWGRbWAcqGGSaalcqWGSbaBaeqaniabggHiLdGccaWLjaGaaCzcaiabcIcaOiabigdaXiabcMcaPaaa@8922@

subject to

∑jhij≤1,i∈{1,...,N}.
 MathType@MTEF@5@5@+=feaafiart1ev1aaatCvAUfKttLearuWrP9MDH5MBPbIqV92AaeXatLxBI9gBaebbnrfifHhDYfgasaacH8akY=wiFfYdH8Gipec8Eeeu0xXdbba9frFj0=OqFfea0dXdd9vqai=hGuQ8kuc9pgc9s8qqaq=dirpe0xb9q8qiLsFr0=vr0=vr0dc8meaabaqaciaacaGaaeqabaqabeGadaaakeaadaaeqbqaaiabdIgaOnaaBaaaleaacqWGPbqAcqWGQbGAaeqaaaqaaiabdQgaQbqab0GaeyyeIuoakiabgsMiJkabigdaXiabcYcaSiabdMgaPjabgIGiolabcUha7jabigdaXiabcYcaSiabc6caUiabc6caUiabc6caUiabcYcaSiabd6eaojabc2ha9jabc6caUaaa@4531@

Thereby, the second index *j *∈ {1, ..., *N*_*i*_} always runs over all *N*_*i *_candidate TIS associated with gene *i*. Thus, the estimation-step for computing the probabilities maximizes the log-likelihood of the models under a given classification, i. e. with fixed *h*_*ij*_. So at least the scores keep constant with the application of that step. Given the probability matrices **P**_*s *_and **P**_*w*_, the classification step only changes an assignment variable if the score can be increased. If no change can improve the score, the algorithm is finished because the previous classification is maintained and nothing will change in any succeeding iteration. Thereby, the above constraint on the assignment variables assures that for any gene at most one candidate TIS is classified as a *strong *one, i. e. has a non-zero assignment variable.

Although closely related, the above clustering scheme is not identical to the well-known EM algorithm as originally proposed in [13]. However it may be viewed as a special variant which implements *deterministic *instead of *probabilistic *assignment variables and which imposes a TIS specific constraint on the assignment variables *h*_*ij*_. This constraint ensures that at most *one *candidate TIS of a putative gene is considered as a *strong *candidate, even in cases where several gene-specific candidates provide positive PWM scores. The constraint implements some task-specific knowledge about the general TIS prediction problem which is important for the performance of the algorithm because it effectively improves the signal-to-noise ratio with respect to estimation of the *strong *TIS model.

The only condition to be met for convergence of the above optimization is that the objective function *F *has to be upper bounded which implies that all entries of the above probability matrices must be non-zero values in order to provide finite scores. This requirement can be achieved by a positional smoothing procedure as described in the following section.

### Positional smoothing

For the above clustering scheme to converge, it is necessary to avoid infinite scores. These may arise from zero probabilities in the stochastic matrices for representation of trinucleotide occurrences in the flanking regions of the TIS candidate sequences.

It can be observed that the occurrences of short nucleotide patterns in TIS sequences show some positional uncertainty [12]. Although certain patterns usually occur with high probability at certain positions, these occurrences can also be observed at neighboring positions to some extent. This implies that there are strong correlations between the occurrences of patterns at neighboring positions. These correlations in turn suggest a simple scheme to cope with vanishing probabilities resulting from the estimation of an inhomogeneous higher order Markov model: to avoid zero probabilities in the estimated models, the counts of neighboring positions may be averaged, i. e. a *positional smoothing *of the corresponding counts may be applied.

Formally, an inhomogeneous *L*-position probability model of order *K *- 1 can be represented by some 4^*K *^× *L *stochastic matrix **P**. Each column of **P **is a probability vector with nonnegative values summing up to one. Thereby an entry *p*_*kl *_of that matrix denotes the probability of the *k*-th *K*-mer to occur at position *l*. Linear smoothing can be realized by post multiplication with an *L *× *L *stochastic matrix **S **which contains shifted versions of the discretized smoothing function. In our case we chose a Gaussian smoothing function with bandwidth parameter *σ *controlling the degree of smoothing. This choice results in the normalized entries

smn=e(−12σ2(m−n)2)∑le(−12σ2(l−n)2)
 MathType@MTEF@5@5@+=feaafiart1ev1aaatCvAUfKttLearuWrP9MDH5MBPbIqV92AaeXatLxBI9gBaebbnrfifHhDYfgasaacH8akY=wiFfYdH8Gipec8Eeeu0xXdbba9frFj0=OqFfea0dXdd9vqai=hGuQ8kuc9pgc9s8qqaq=dirpe0xb9q8qiLsFr0=vr0=vr0dc8meaabaqaciaacaGaaeqabaqabeGadaaakeaacqWGZbWCdaWgaaWcbaGaemyBa0MaemOBa4gabeaakiabg2da9maalaaabaGaemyzau2aaWbaaSqabeaadaqadaqaaiabgkHiTmaalaaabaGaeGymaedabaGaeGOmaidcciGae83Wdm3aaWbaaWqabeaacqaIYaGmaaaaaSGaeiikaGIaemyBa0MaeyOeI0IaemOBa4MaeiykaKYaaWbaaWqabeaacqaIYaGmaaaaliaawIcacaGLPaaaaaaakeaadaaeqaqaaiabdwgaLnaaCaaaleqabaWaaeWaaeaacqGHsisldaWcaaqaaiabigdaXaqaaiabikdaYiab=n8aZnaaCaaameqabaGaeGOmaidaaaaaliabcIcaOiabdYgaSjabgkHiTiabd6gaUjabcMcaPmaaCaaameqabaGaeGOmaidaaaWccaGLOaGaayzkaaaaaaqaaiabdYgaSbqab0GaeyyeIuoaaaaaaa@5442@

with *l*, *m*, *n *∈ {1 ... *L*}. As indicated above, the smoothed matrix of probabilities smn=e(−12σ2(m−n)2)∑le(−12σ2(l−n)2)     (2)
 MathType@MTEF@5@5@+=feaafiart1ev1aaatCvAUfeBSjuyZL2yd9gzLbvyNv2CaerbwvMCKfMBHbqedmvETj2BSbqee0evGueE0jxyaibaieYdOi=BH8vipeYdI8qiW7rqqrFfpeea0xe9Lq=Jc9vqaqpepm0xbbG8FasPYRqj0=yi0lXdbba9pGe9qqFf0dXdHuk9fr=xfr=xfrpiWZqaaeaabiGaaiaacaqabeaabeqacmaaaOqaaiaadohadaWgaaWcbaGaamyBaiaad6gaaeqaaOGaeyypa0ZaaSaaaeaacaWGLbWaaWbaaSqabeaadaqadaqaaiabgkHiTmaalaaabaGaaGymaaqaaiaaikdaiiGacqWFdpWCdaahaaadbeqaaiaaikdaaaaaaSGaaiikaiaad2gacqGHsislcaWGUbGaaiykamaaCaaameqabaGaaGOmaaaaaSGaayjkaiaawMcaaaaaaOqaamaaqababaGaamyzamaaCaaaleqabaWaaeWaaeaacqGHsisldaWcaaqaaiaaigdaaeaacaaIYaGae83Wdm3aaWbaaWqabeaacaaIYaaaaaaaliaacIcacaWGSbGaeyOeI0IaamOBaiaacMcadaahaaadbeqaaiaaikdaaaaaliaawIcacaGLPaaaaaaabaGaamiBaaqab0GaeyyeIuoaaaGccaWLjaGaaCzcamaabmaabaGaaGOmaaGaayjkaiaawMcaaaaa@542A@ is simply obtained from the matrix product

smn=e(−12σ2(m−n)2)∑le(−12σ2(l−n)2)     (2)
 MathType@MTEF@5@5@+=feaafiart1ev1aaatCvAUfeBSjuyZL2yd9gzLbvyNv2CaerbwvMCKfMBHbqedmvETj2BSbqee0evGueE0jxyaibaieYdOi=BH8vipeYdI8qiW7rqqrFfpeea0xe9Lq=Jc9vqaqpepm0xbbG8FasPYRqj0=yi0lXdbba9pGe9qqFf0dXdHuk9fr=xfr=xfrpiWZqaaeaabiGaaiaacaqabeaabeqacmaaaOqaaiaadohadaWgaaWcbaGaamyBaiaad6gaaeqaaOGaeyypa0ZaaSaaaeaacaWGLbWaaWbaaSqabeaadaqadaqaaiabgkHiTmaalaaabaGaaGymaaqaaiaaikdaiiGacqWFdpWCdaahaaadbeqaaiaaikdaaaaaaSGaaiikaiaad2gacqGHsislcaWGUbGaaiykamaaCaaameqabaGaaGOmaaaaaSGaayjkaiaawMcaaaaaaOqaamaaqababaGaamyzamaaCaaaleqabaWaaeWaaeaacqGHsisldaWcaaqaaiaaigdaaeaacaaIYaGae83Wdm3aaWbaaWqabeaacaaIYaaaaaaaliaacIcacaWGSbGaeyOeI0IaamOBaiaacMcadaahaaadbeqaaiaaikdaaaaaliaawIcacaGLPaaaaaaabaGaamiBaaqab0GaeyyeIuoaaaGccaWLjaGaaCzcamaabmaabaGaaGOmaaGaayjkaiaawMcaaaaa@542A@ = **P**·**S**.     (3)

Because **P **and **S **are both stochastic matrices, i. e. they have unit column sums, the resulting smn=e(−12σ2(m−n)2)∑le(−12σ2(l−n)2)     (2)
 MathType@MTEF@5@5@+=feaafiart1ev1aaatCvAUfeBSjuyZL2yd9gzLbvyNv2CaerbwvMCKfMBHbqedmvETj2BSbqee0evGueE0jxyaibaieYdOi=BH8vipeYdI8qiW7rqqrFfpeea0xe9Lq=Jc9vqaqpepm0xbbG8FasPYRqj0=yi0lXdbba9pGe9qqFf0dXdHuk9fr=xfr=xfrpiWZqaaeaabiGaaiaacaqabeaabeqacmaaaOqaaiaadohadaWgaaWcbaGaamyBaiaad6gaaeqaaOGaeyypa0ZaaSaaaeaacaWGLbWaaWbaaSqabeaadaqadaqaaiabgkHiTmaalaaabaGaaGymaaqaaiaaikdaiiGacqWFdpWCdaahaaadbeqaaiaaikdaaaaaaSGaaiikaiaad2gacqGHsislcaWGUbGaaiykamaaCaaameqabaGaaGOmaaaaaSGaayjkaiaawMcaaaaaaOqaamaaqababaGaamyzamaaCaaaleqabaWaaeWaaeaacqGHsisldaWcaaqaaiaaigdaaeaacaaIYaGae83Wdm3aaWbaaWqabeaacaaIYaaaaaaaliaacIcacaWGSbGaeyOeI0IaamOBaiaacMcadaahaaadbeqaaiaaikdaaaaaliaawIcacaGLPaaaaaaabaGaamiBaaqab0GaeyyeIuoaaaGccaWLjaGaaCzcamaabmaabaGaaGOmaaGaayjkaiaawMcaaaaa@542A@ is also a stochastic matrix.

By inspection of the smoothing matrix **S**, it is easy to see that the smoothing is inhomogeneous over the range of positions: the degree of smoothing decreases at the boundaries of the sequence window because there, the effective number of neighboring positions used for local averaging of probabilities is actually smaller. In order to eliminate this kind of boundary effect we neglect the weights at the first and last three positions of the resulting PWM. In that way, boundary effects can be excluded for small *σ*-values, relevant for the TIS prediction task. Therefore, the number of sequence positions which are used for scoring of the TIS candidates is actually smaller than the number of sequence positions which are used for estimating the model probabilities.

Note, that *pseudo counts*, which can also be used to avoid vanishing probabilities, provide another special case of linear, i. e. matrix product based, smoothing. That special case is achieved if the smoothing matrix **S **is assembled from the identity matrix with a constant pseudo count offset added to all matrix entries. The resulting matrix has to be scaled to provide unit column sums. Because the above Gaussian smoothing is more local than the pseudo-count smoothing, it is preferable if correlations only exist between close-by positions.

We would like to point out that the smoothing of probabilities can also be realized by means of an explicit smoothing prior on the above stochastic matrices. A suitable smoothing prior on probability vectors together with a Bayesian scheme for automatic adaptation of the corresponding smoothing parameters has for instance been proposed in [14]. Alternatively, a suitable cross-validation scheme which measures the performance on hold-out data, may be used to automatically control the smoothing. In the following we present a cross-validation procedure for adaptation of the smoothing parameter *σ *which makes use of the particular constraints of the TIS-prediction setup.

### 1 Automatic adaptation of the smoothing parameter

In order to provide an automatic adaptation of the Gaussian smoothing for a particular genome, the smoothing parameter *σ *is adjusted according to a special cross-validation procedure. For that purpose we measure the performance of the model with respect to discrimination between all *strong *TIS candidates (class 1) and their associated *weak *TIS candidates (class 2). For discrimination we only consider *weak *TIS which are among the candidates of a putative gene with a *strong *TIS. In that way we exploit the fact that if a *strong *candidate is actually a *true *TIS, then the associated *weak *candidates must be *false *TIS. Therefore, it makes sense to measure the discriminative power of the model with regard to the above two classes 1 and 2, respectively. As a performance measure we use the *area under curve *(AUC) criterion with respect to the receiver operating characteristics (ROC) curve [15,16]. The AUC measures the performance of a binary classifier under varying costs for the false positive and false negative misclassification and therefore it provides a suitable criterion for unbalanced setups, where the number of negative examples may be much higher than the number of positive examples. For estimation of the AUC we perform a 10-fold cross-validation: the TIS examples are divided in 10 subsets with an equal proportion of the two classes in each subset. Once a subset is used for testing the model, based on the corresponding PWM test scores, the other nine subsets are used for estimation of the underlying PWM. The average test AUC of all ten subsets is used to rate the current *σ*-value. In that way, the AUC performance is computed for a set of 16 possible values according to the sampling *σ *∈ {0.25, 0.3, 0.35, ..., 1.0} and finally the value with maximum average AUC is selected.

For an overall optimization of all model parameters, an iterative scheme for repeated adaptation of the smoothing parameter is required. The overall scheme proceeds in the following way:

1. *Initialization: *Set *σ *= 0.5.

2. *Clustering: *Run the Estimation-Classification algorithm with current *σ*, as described in the previous section.

3. *Cross-validation: *Select *σ *∈ {0.25, 0.3, 0.35, ..., 1.0} according to a maximum average AUC, as described above.

4. *Stopping Criterion: *Abort, if *σ *has not been changed at the previous step. Continue with step 2 using new *σ*, otherwise.

## Results

### Datasets

Despite the growing number of sequenced bacterial genomes, evaluation of prokaryotic gene prediction is still difficult because most available annotations are based on computational predictions which are likely to contain errors, especially with respect to the exact location of translation starts. Therefore, currently the most important sources of information for evaluation of prokaryotic gene prediction are the EcoGene [17] database and the Link dataset [18]. EcoGene provides annotations for 854 genes from *Escherichia coli *K-12 [GenBank:NC_000913] [19] with N-termini verified by protein sequencing. The Link dataset largely is a subset of the former and includes 195 genes.

In addition to the *E. coli *genome we also studied the performance of our method on the genome of *Bacillus subtilis*, *Pseudomonas aeruginosa*, *Burkholderia pseudomallei *and *Ralstonia solanacearum*. Among these species the last three ones are known to have *high-G+C genomes *with a G+C content exceeding 66%. For *Bacillus subtilis *[GenBank:NC_000964] [20], we used all *non-y *genes of the GenBank annotation for comparison. These genes are experimentally characterized and have verified start sites [21,8]. Additionally we evaluated TICO on a small subset of 58 genes verified by comparison to the closely related organism *B. halodurans *[21]. The verified datasets of *E. coli *and *B. subtilis*, as described above, were obtained from the web site of the Center of Theoretical Biology (CTB) [22] at Peking University. For the evaluation of our algorithm on high-G+C genomes we first chose *Pseudomonas aeruginosa *PA01 with a G+C content of 66.6%. After the genome of *P. aeruginosa *was completely sequenced and published [23] the analysis has been continued by the Pseudomonas Community Annotation Project (PseudoCAP) [24] in order to improve the quality of the first annotation. We used the latest version of the sequence and the annotation from May 2005 containing 5647 annotated genes. From our analysis we excluded all genes which have been labeled as *unknown, uncharacterized *or *hypothetical *in the corresponding annotation table. In that way, 3281 carefully annotated genes with an assigned function were considered for the evaluation.

In addition, we investigated the prediction performance on two other high-G+C genomes: *Burkholderia pseudomallei *strain K96243 chromosome 1 [GenBank:NC_006350] and chromosome 2 [GenBank:NC_006351] [25] with a G+C content of 67.7% and 68.56%, respectively and *Ralstonia solanacearum *chromosome [GenBank:NC_003295] and megaplasmid [GenBank:NC_003296] [26] with a G+C content of 67.04% and 66.86%, respectively. Both genomes are assumed to contain a large number of repeats, inserts, prophages and putative alien genes, which are likely to complicate the prediction for computational methods. The pathogen bacterium *B. pseudomallei *causing melioidosis has been sequenced and annotated by the Sanger Trust Institute [27]. The GenBank annotation is based on comparative analysis using the Artemis Comparative Tool. The plant pathogen bacterium *R. solanacearum *is a model organism for the analysis of pathogenicity and it has been subject to intensive studies based on biochemical and genetical analysis [26]. The GenBank annotation which we used as a reference is based on the prediction of the program FrameD [28], explicitly trained for this organism, in combination with comparative methods.

Note that the TIS locations of *B. subtilis*, *P. aeruginosa*, *B. pseudomallei *and *R. solanacearum *have not been verified experimentally, even though, in many cases the gene products are well characterized or gene starts have been annotated manually.

### Performance

We first study the performance of our method, using the automatic adaptation of the smoothing parameter, as described in the previous sections. The corresponding cross-validation procedure (see section »Automatic Adaptation of the Smoothing Parameter«) has been implemented as an extension of the tool TICO [11] where it is used by default. For initial prediction of the TIS locations we used GLIMMER2.02 [1] and measured the improvement which was obtained from the application of our algorithm. The region for searching suitable candidate TIS around the initial GLIMMER-predicted gene start was chosen to range from 250 nt upstream to 250 nt downstream of the initial start. The extracted sequence windows for estimation of the trinucleotide probabilities were chosen to range from 30 nt upstream to 30 nt downstream of each candidate TIS.

With respect to the genome of *E. coli*, GLIMMER2.02 found 846 (99.1%) of the 854 verified genes in EcoGene. For these 846 detected genes only 63.2% of the predicted TIS locations were correct. Using TICO for post-processing of the GLIMMER predictions, this rate was increased by 31%. Also for *B. subtilis *we achieved a considerable improvement of the initial GLIMMER prediction with a resulting increase of 28.1%. The improvement for the high-G+C genomes was not as large for all species. While for *P. aeruginosa *we still achieved an increase of 27.4%, the improvement for *R. solanacearum *was 23.4% (chromosome) and 21.2% (plasmid), respectively and for *B. pseudomallei *it was 16.4% (chromosome 1) and 18.1% (chromosome 2), respectively. These rates show that our method provides a valuable tool for the annotation of prokaryotic genomes. Because the underlying algorithm performs in a completely unsupervised manner, it especially applies to the case where no knowledge exists about the signals, initiating the translation. Obviously, this situation is usually encountered for newly sequenced genomes. Another problem, well-suited for the application of our algorithm, is the reannotation of published genomes. As mentioned above, the annotation of many genes is based on results obtained from conventional gene finders. Therefore, the annotation of the TIS locations is likely to contain errors. Comparing the GenBank annotation of *E. coli *K-12 with the experimentally verified data from the EcoGene dataset, only 89.8% of these TIS are annotated correctly. We initialized our algorithm with the GenBank annotation and could increase the accuracy of the TIS annotation to a rate of 95.1% correct sites.

#### User-selected smoothing parameter

**Figure 1:**
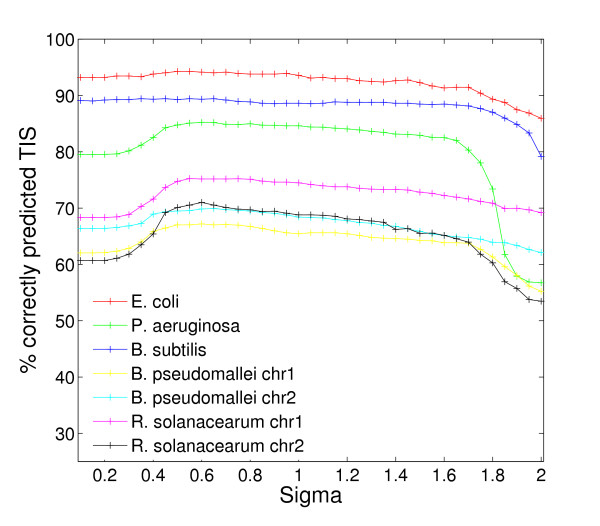
**Dependence on user-selected smoothing parameter**. Performance of TICO on the genomes of *E. coli*, *B. subtilis*, *P. aeruginosa*, *B. pseudomallei *and *R. solanacearum *for a user-selected smoothing parameter varying according to *σ *= {0.1, 0.15, ..., 2.0}. Performance is measured in percentage of correctly predicted TIS as compared with the respective reference dataset (see section »Datasets« for details).

As an alternative to the automatic adaptation of the smoothing parameter sigma (*σ*), TICO also allows the user to specify the degree of smoothing manually, like in the first version of that tool [11]. In order to investigate the dependence of the prediction performance on a user-selected parameter, we applied the algorithm on the above three genomes, with a fixed smoothing parameter selected according to *σ *= 0.1, 0.15, 0.2, ..., 2.0. The prediction performance was measured on the corresponding test sets as described above. The resulting prediction rates are shown in figure 1. Although the performance of TICO is rather stable over a wide sigma range, the performance significantly decreases for smaller values below 0.4. This effect can especially be observed for the high-G+C genomes of *Ralstonia*, *Pseudomonas *and *Burkholderia*. For sigma-values above 1.5 an »over-smoothing« effect becomes apparent for all genomes.

Comparing the best results which can be achieved for a fixed sigma, with those of the automatic adaptation, the observed differences are very small. In most cases the best rate of the user-selected smoothing was slightly above the rate of the automatic procedure. For *E. coli *the difference was negligible with rates 94.3% (*σ *= 0.5) and 94.2% (*σ *= 0.45) for user and automatic mode, respectively. For *B. subtilis *the best rate (84.4%) and sigma (*σ *= 0.45) were the same in both cases. Also for *P. aeruginosa *the corresponding rates were identical, with the best user-selected sigma (*σ *= 0.6) slightly above the automatic selection (*σ *= 0.55). The only deviation which is noteworthy at all, we observed for *R. solanacearum *on the plasmid with rates 71.0% (*σ *= 0.6) and 70.1% (*σ *= 0.45) for the user and automatic mode, respectively. In that case, the automatic selection seemed to result in a slight under-smoothing. In all other cases the difference between rates was at most 0.3%. Comparing the small differences between the best-case user selection and the automatic selection with those differences which can be obtained for a worst-case user-selection (compare figure 1), we strongly recommend to use the automatic adaptation which is also the default setting in TICO.

We like to emphasize that in practice, it would not be possible to determine the best user-selected sigma, because usually no reliable reference annotation would be available. Therefore, evaluation of a hypothetical user-selection is somewhat artificial and the corresponding best rates can just be viewed as an upper bound for the performance of our algorithm. Consequently, these rates are not used in the following comparison.

### Comparison with other tools for improvement of TIS predictions

To our knowledge, currently three other tools are available for improving predictions of prokaryotic TIS. In a recent publication [5] these tools have been compared for their performance on reliable test data from *E. coli *and *B. subtilis*. Although these tools perform well on the utilized test data, it is important to realize that the corresponding models include specific assumptions about the statistical nature of prokaryotic TIS. In RBSfinder [10] gene starts are relocated by iteratively scoring potential RBS near annotated start codons predicted by a gene finder. Scoring is based on a probabilistic model with some of the parameters relying on prior assumptions about sequence statistics. In addition several empirical thresholds have to be adjusted which finally control the performance of the score-based selection of TIS locations.

In MED-Start [5] also a probabilistic model for prokaryotic TIS is utilized for iteratively rescoring the candidate TIS. While most of the model parameters are optimized in an unsupervised manner, the length of Shine-Dalgarno (SD) [29] motifs has to be specified, using a length 5 default value. In addition, the maximum number of SD motifs to be considered is pre-specified. In [5] it remains unclear how automatic selection of the actual number of SD motifs utilized for a specific genome has been realized in MED-Start. In GS-Finder [4] several sequence features from the flanking regions of candidate TIS are utilized for an iterative relocation procedure. While most features are used in an unsupervised manner the method also relies on some prior assumptions about start codon usage and about the distribution of the distance between the leftmost candidate start codon and the true TIS.

In contrast, our method does not require any specific model assumptions about prokaryotic TIS. Only rather general assumptions about the TIS relocation problem can be found to be implemented in the default values of TICO. These defaults concern the search range for alternative TIS around the initial predictions and the size of the sequence window for scoring the TIS candidates. We would like to point out that this kind of assumptions can be found in all post-processors considered here and that these assumptions should be distinguished from assumptions about TIS related sequence content features. These specific assumptions which can be found in other post-processors as indicated above, have been avoided in TICO because they may not hold across different species with different sequence characteristics. Therefore our algorithm performs without any specification of parameters concerning the composition and location of SD motifs or the usage of start codons. This is of particular importance for genomes with a lower information content in the SD motifs, as is usually the case in high-G+C genomes [30]. Furthermore, no empirical thresholds which could imply a severe bias, are involved in the analysis. For the scoring of potential TIS we only consider smoothed trinucleotide probabilities in the flanking regions of the corresponding candidate start codons. The only special parameter of our method which has influence on the performance, is the degree of positional smoothing as adjusted by the parameter sigma (*σ*). With the default setting, in the latest version of TICO this parameter is adapted automatically (see section »Automatic Adaptation of the Smoothing Parameter«).

In order to compare our algorithm with the other three tools on the test data described above, we used the predictions of the tool GLIMMER2.02 for initial annotation of the coding regions in all cases. The results of GLIMMER, TICO, RBSfinder, MED-Start and GS-Finder are summarized in Table 1. The prediction rates of GS-Finder, MED-Start and TICO are comparable on the datasets of *E. coli *and *B. subtilis*. With respect to the rate of correctly predicted TIS from the reference set, the maximum difference between these tools is 3.9%. The improvement of the GLIMMER-prediction is at least 26.6%. The rates achieved by RBSfinder are about 10% below the results of the former tools. For *E. coli *the best rates are 94.2% (TICO) on the EcoGene dataset and 95.4% (MED-Start) on the Link dataset. On the large dataset of *B. subtilis *the best rate was 89.4% (TICO), on the small subset the best result was 94.8% (GS-Finder, MED-Start). Note that the rates on the small dataset of *B. subtilis *containing 58 sequences are not as representative as the rates on the larger dataset in terms of statistical significance.

**Figure 2:**
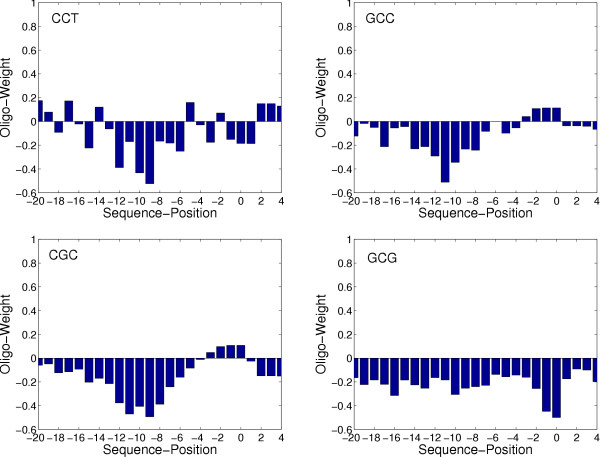
**Exemplary trimer weights calculated by TICO**. Positional weight matrix (PWM) values resulting from our algorithm for four exemplary trimers in the flanking regions of the TIS. Position 0 denotes the translation start. Selected trimers correspond to the most frequent subwords in the putative SD motifs determined by MED-Start [5] for *P. aeruginosa *(CCTGG, GCGCC, GCCTG, CGCCG and CGGCG). Negative weights indicate that trimer occurrences at the corresponding positions are untypical for *strong *TIS candidates.

On the high-G+C genomes only TICO and GS-Finder provide good results with a maximum difference of 5.3% between both tools. The maximum prediction rate on *P. aeruginosa *is 85.2% (TICO), on *B. pseudomallei *it is 69.6% (TICO) and 67.5% (GS-Finder) for chromosome 1 and 2, respectively and on *R. solanacearum *the maximum rate is 74.9% (TICO) and 70.1% (TICO) for the chromosome and the plasmid, respectively. The performance of RBSfinder is about 15 to 20 percent below these rates. MED-Start completely failed on these genomes. The best rate, MED-Start achieved on the high-G+C genomes was 6.0%. Only 3.6% of the predicted TIS were in correspondence with the PseudoCAP annotation. To explain these results, we evaluated the SD motifs suggested by MED-Start. The following five motifs were proposed to be related with the translation initiation in *P. aeruginosa*: CCTGG, GCGCC, GCCTG, CGCCG and CGGCG. Our first impression was that these motifs are very different from patterns of known SD motifs, which are usually described by the regular expression RGGRGGTGAT (R = A or G) [29]. Comparing the MED-Start motifs with the resulting PWM model of our algorithm, we found most of the trimers contained in the motifs to be associated with high negative weights at the respective position. Nearly all subwords of the MED-Start motifs (like TGG, CCT, CGG, GCC, ...) are among the 15 trimers with the highest negative weight. In other words, the pattern MED-Start »learned« to be related with a TIS, may be most frequent in the upstream region of TIS candidates, but it is by no means a TIS signal. Figure 2 shows the resulting weights (PWM values) of our algorithm for those four trimers occurring most frequent in the MED-Start motifs. As indicated above, these trimers have negative weights almost everywhere and therefore they are unlikely to provide characteristic features of real TIS.

## Conclusion

We introduced a completely unsupervised approach to the problem of TIS prediction in prokaryotic genomes. We showed that our clustering algorithm provides a valuable tool for genome annotation and reannotation, respectively. We found that even on high-G+C genomes, a considerable improvement of TIS prediction accuracy can be achieved by means of our algorithm. In summary, we expect our method to offer advantages for the analysis of new genomes where nothing is known a priori about the associated Shine-Dalgarno motif or other specific sequence features of the underlying gene starts. The latest version of the corresponding tool is publicly available at [31].

## Authors' contributions

PM had the main idea of the algorithm, implemented the clustering scheme and drafted most parts of the manuscript. MT contributed biological expertise, implemented the tool TICO, performed the experimental evaluation and drafted substantial parts of the manuscript. Both authors read and approved the final manuscript.

## Figures and Tables

**Table 1:** TIS prediction accuracy of our algorithm (TICO) in comparison with other post-processing tools. RBSfinder [10], GS-Finder [4] and MED-Start [5] were used as post-processors on the same GLIMMER2.02-prediction as TICO. Accuracy was measured in percent of TIS that were predicted correctly with respect to reference annotations. Datasets are explained in detail in section »Results«.

**Dataset**	**total number**	**%detected ORFs**	**% correctly predicted TIS**				
			GLIMMER	MED-Start	TICO	GS-Finder	RBSfinder
EcoGene	854	99.3	63.2	92.0	**94.2**	90.3	81.9
Link	195	100	66.7	**95.4**	94.9	92.3	80.0
Bsub	1248	98.6	61.3	89.2	**89.4**	87.9	78.5
	58	98.3	69.0	**94.8**	91.4	**94.8**	82.8
PseudoCAP	3281	97.5	57.8	3.6	**85.2**	83.6	67.7
*R. solanac*. chr	3440	97.2	51.5	5.0	**74.9**	71.4	56.8
*R. solanac*. plasmid	1676	97.0	48.9	6.0	**70.1**	66.2	55.5
*B. pseudom*. chr1	3399	97.7	53.2	5.5	**69.6**	64.3	53.3
*B. pseudom*. chr2	2329	97.7	48.9	4.7	67.0	**67.5**	52.1
